# Calibration and Performance Evaluation of Cost-Effective Capacitive Moisture Sensor in Slope Model Experiments

**DOI:** 10.3390/s24248156

**Published:** 2024-12-20

**Authors:** Muhammad Nurjati Hidayat, Hemanta Hazarika, Haruichi Kanaya

**Affiliations:** 1 Graduate School of Engineering, Kyushu University, Fukuoka 819-0395, Japan; hidayat.muhammad.606@s.kyushu-u.ac.jp; 2 Graduate School of Information Science and Electrical Engineering, Kyushu University, Fukuoka 819-0395, Japan; kanaya@ed.kyushu-u.ac.jp

**Keywords:** soil moisture sensor, water content, sensor calibration, slope model, internet of things

## Abstract

Understanding the factors that contribute to slope failures, such as soil saturation, is essential for mitigating rainfall-induced landslides. Cost-effective capacitive soil moisture sensors have the potential to be widely implemented across multiple sites for landslide early warning systems. However, these sensors need to be calibrated for specific applications to ensure high accuracy in readings. In this study, a soil-specific calibration was performed in a laboratory setting to integrate the soil moisture sensor with an automatic monitoring system using the Internet of Things (IoT). This research aims to evaluate a low-cost soil moisture sensor (SKU:SEN0193) and develop calibration equations for the purpose of slope model experiment under artificial rainfall condition using silica sand. The results indicate that a polynomial function is the best fit, with a coefficient of determination (R^2^) ranging from 0.918 to 0.983 and a root mean square error (RMSE) ranging from 1.171 to 2.488. The calibration equation was validated through slope model experiments, with soil samples taken from the models after the experiment finished. Overall, the moisture content readings from the sensors showed approximately a 12% deviation from the actual moisture content. The findings suggest that the cost-effective capacitive soil moisture sensor has the potential to be used for the development of landslide early warning system.

## 1. Introduction

Landslides, particularly those triggered by rainfall, pose a significant threat to human life and infrastructure worldwide. The global incidence of landslides is alarming, with thousands of events reported each year [1], often resulting in catastrophic damage and loss of life. Japan, with its mountainous terrain and frequent typhoons, faces a recurring threat of landslides caused by rainfall. Saito [2] reports numerous such events each year, worsened by intense seasonal rains and typhoons. These landslides cause destruction to infrastructures and significant economic losses and disruptions [3]. Therefore, it is crucial to have effective monitoring and early warning systems in place in these regions. Understanding the factors that contribute to slope failures, such as rainfall patterns and soil saturation levels, is essential for the development of such systems. Research indicates that accurate prediction and timely alerts can mitigate the impact of these disasters [4,5]. Consequently, there is a growing focus on enhancing the tools and techniques utilized for monitoring slopes at risk of landslides.

To comprehend the mechanisms of slope failure, many researchers have conducted small-scale slope model experiments within laboratory settings [6,7,8,9,10]. These experiments aim to replicate real-world conditions to study the behavior of slopes under various scenarios. Common equipment used in these studies includes inclinometers, which measure the angle of slope movements, and pore pressure transducers, which monitor water pressure within the soil. Additionally, soil moisture sensors are crucial for detecting changes in soil water content, a key factor in slope stability [11,12,13,14]. These sophisticated instruments provide detailed insights into the processes leading to slope failure, contributing significantly to our understanding of landslide dynamics. However, the high cost and complexity of these instruments limit their widespread application, especially in resource-constrained settings. Therefore, there is a need for more accessible and cost-effective monitoring solutions. Although some progress has been made in this area [14,15,16,17], many existing systems remain impractical for widespread application due to challenges related to affordability, scalability, need for expertise, and maintenance in remote or developing regions. These limitations underscore the ongoing inadequacies in landslide monitoring systems, particularly in susceptible locations that would benefit from more long-term solution.

With the current advancements in technology, the development of low-cost sensors integrated with the Internet of Things (IoT) presents a promising solution. IoT technology enables the remote collection and transmission of data, making it possible to monitor slope failure vulnerability in multiple sites simultaneously and in real time [18,19,20]. One notable example is the SKU:SEN0193, a low-cost capacitive moisture sensor that offers a feasible alternative for widespread monitoring and has been widely studied for agricultural purposes [21,22,23,24]. This sensor is not only affordable but also capable of providing real-time data. Recent studies on alternative moisture sensing materials have highlighted advancements in sensor stability and sensitivity across diverse environmental conditions, emphasizing the growing potential of innovative sensor technologies for monitoring applications [25,26]. In the context of landslide monitoring and mitigation, SKU:SEN0193 has the potential to be used effectively in landslide early warning systems. Moreover, integrating this soil moisture sensor with IoT platforms allows for continuous monitoring and data analysis, enhancing the effectiveness of landslide prediction models. This approach not only reduces costs but also improves the accessibility of landslide monitoring technologies.

To ensure the accuracy and reliability of this low-cost moisture sensor, calibration on specific soil types and properties is essential, as the calibration results significantly influence sensor readings, making calibration a critical step [22,24,27]. This research aims to evaluate the performance of low-cost capacitive moisture sensors (SKU:SEN0193) by developing calibration equations to predict soil moisture content. To verify the results, the moisture sensors were tested in slope model experiments under laboratory condition in different rainfall intensities. After the completion of the slope model experiment, the moisture contents detected by the sensors were compared against the actual soil moisture contents. The validation of calibration equations through slope model experiments shows the effectiveness of the moisture sensors in delivering accurate data, thus supporting cost-effective solutions for a landslide early warning system.

## 2. Materials and Methods

### 2.1. Data Acquisition

**Figure 1:**
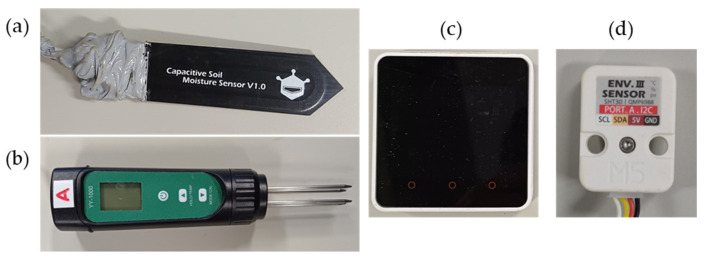
Equipment used: (**a**) moisture sensor, (**b**) soil moisture meter, (**c**) M5Stack Core2, and (**d**) ENV III sensor.

Six low-cost capacitive soil moisture sensors SKU:SEN0193 from DFRobot were used in this study. Each sensor operates within a voltage input range of 3.3 to 5.5 V, measures 9.8 × 2.3 cm (L × W), and weights 15 grams, making it compatible with low-power microcontrollers [28]. Its surface is coated with a corrosion-resistant material, enhancing its durability. To protect the electronic components of the moisture sensors from short circuits, oxidation, and physical damage during experiments, the electronic parts were covered with silicone, making them waterproof. From the following, the term moisture sensor is used, instead of SKU:SEN0193. In addition, two Slyfox soil moisture meters were used to measure the soil temperature. This commercial device, designed specifically for agricultural applications, incorporates a temperature measurement feature for soil assessment [29]. Lastly, an ENV III environmental sensor from M5Stack was employed to measure room temperature, humidity, and atmospheric pressure. The ENV III sensor is capable of measuring temperatures ranging from −40 to 120 °C, relative humidity from 10 to 90%, and air pressure from 300 to 1100 hPa. The equipment used for this study is shown in Figure 1.

**Figure 2:**
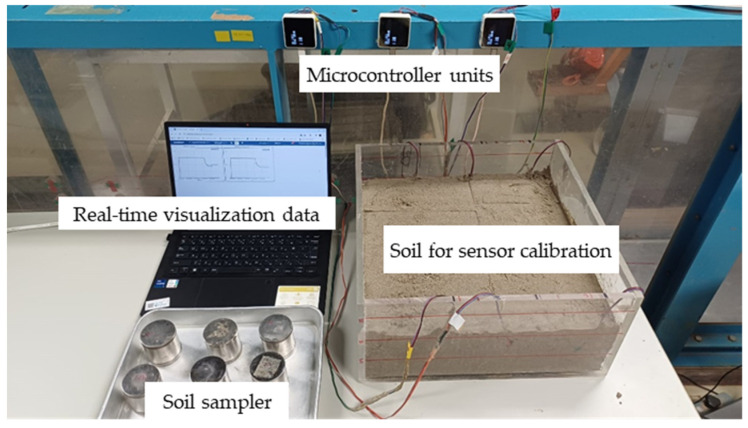
Overview of calibration experiment setup.

**Figure 3:**
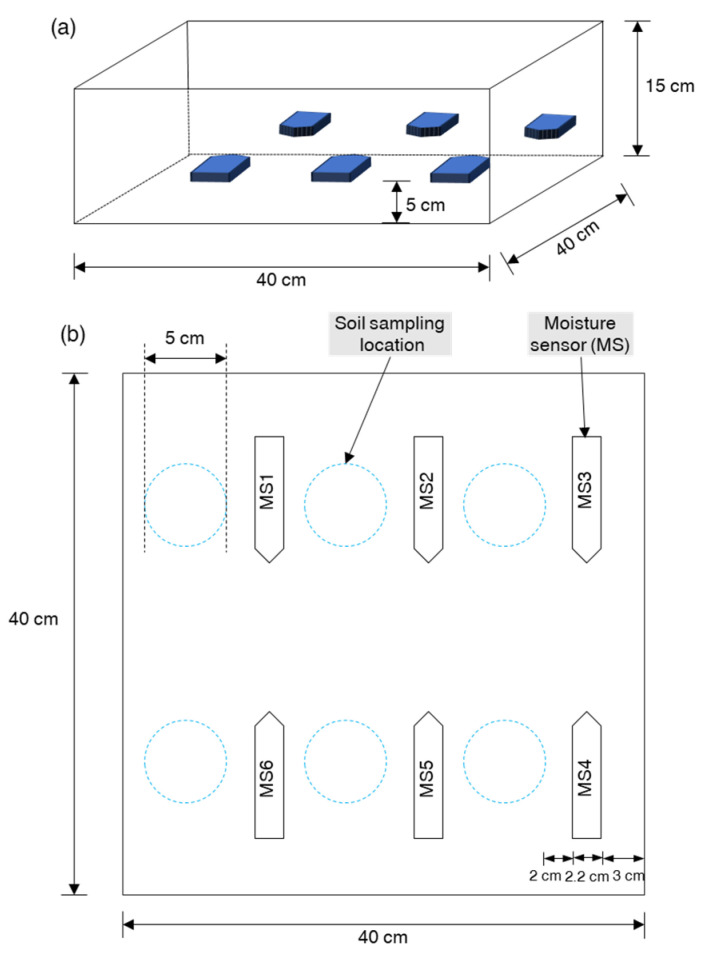
Schematic of the soil box for sensor calibration: (**a**) 3D view of soil box and six soil moisture sensors, and (**b**) soil samples were collected from the blue dashed line area near the moisture sensors to measure actual water content using a soil sampler.

The moisture sensor and ENV III were connected to the M5Stack Core2, serving as a microcontroller unit for aggregating and transmitting data to a cloud server via the internet. The M5Stack Core2 has dimensions of 5.4 cm × 5.4 cm × 1.6 cm (L × W × H) and is equipped with a 390 mAh:3.7 V built-in battery. It features a dual-core Xtensa^®^ 32-bit 240 MHz processor, onboard 16 MB Flash and 8 MB PSRAM, and a USB Type-C interface for charging, program downloading, and serial communication. The M5Stack Core2 was programmed using the Arduino programming language. To monitor and download data from the cloud server, an open-source Internet of Things (IoT) data visualization tool, Ambient (https://ambidata.io/, accessed on 29 February 2024) was utilized. The experiment setup is presented in (Figure 2), while the component and total cost for the study was around US$260 (Table 1). Schematic of the soil box for sensor calibration presented in Figure 3.

### 2.2. Soil Properties and Calibration Process

**Figure 4:**
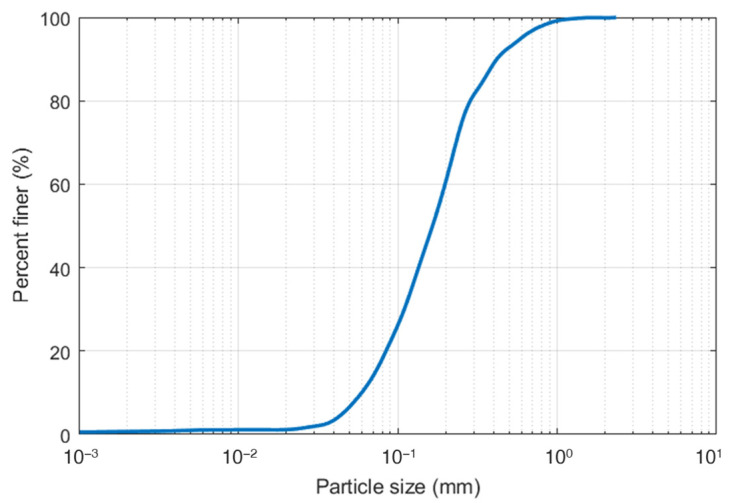
Particle size distribution of K7 sand.

Commercially available silica sand grade 7 was used for this research, hereafter referred to as K7 sand. This sand was chosen because it is frequently utilized in slope model experiments to examine the failure mechanisms associated with rainfall-induced landslides, particularly in scenarios where sandy soils play a critical role in the triggering process [30,31,32,33]. The soil particle size distribution and properties are presented in Figure 4 and Table 2.

To achieve precise calibration and ensure the accuracy of the moisture sensor, a calibration experiment was carefully designed. Upon completion, the output was the calibration equation for each soil moisture sensor. To verify these calibration equations, three slope model experiments with different rainfall intensities were conducted. In the slope model experiments, the raw data from the soil moisture sensors were converted using the calibration equation to measure the soil moisture content. Therefore, the calibration process was designed according to the slope model experiment, since the sensor measurements during calibration are strongly influenced by sample preparation and calibration protocols [34,35]. The calibration experiment involves ten steps:

1. The dimensions of the soil box were measured, and the required amount of soil was determined. Additionally, the weight of soil for two layers was calculated. The soil box was then marked at heights of 5 cm and 10 cm for the first and second layers, respectively.
2. Water equivalent to 5% of the weight of the soil was mixed to the oven-dried soil.
3. Half of the soil prepared in Step 2 was placed in the soil box to create the first layer, which was compacted to match the height of the first layer.
4. Six moisture sensors were placed on top of the first soil layer. The moisture sensors were positioned at an adequate distance from one another to prevent any potential interference.
5. The remaining soil–water mixture prepared in Step 2 was placed and compacted to match the height of the second layer.
6. The soil box was covered with a plastic sheet to minimize potential moisture evaporation and the soil sample was left to homogenized for 20 to 40 min.
7. The moisture sensors recorded three measurements (5 min each). The average value of these measurements was used for the analysis of moisture sensor calibration.
8. Both soil and room temperatures were recorded.
9. Soil samples were collected from locations near the moisture sensors, and the actual soil moisture content was measured.
10. Steps 2 through 9 were repeated, increasing the water incrementally by 5% until the soil sample indicates saturation conditions.

To fit and evaluate both linear and non-linear regression models, the programming language and numeric computing environment MATLAB version R2023b was utilized. The first model was linear, the second was logarithmic and the quadratic was considered as polynomial in the third model. The best-fitted model was determined based on its high coefficient of determination (R^2^) and low root mean square error (RMSE).

### 2.3. Slope Model Experiment

**Figure 5:**
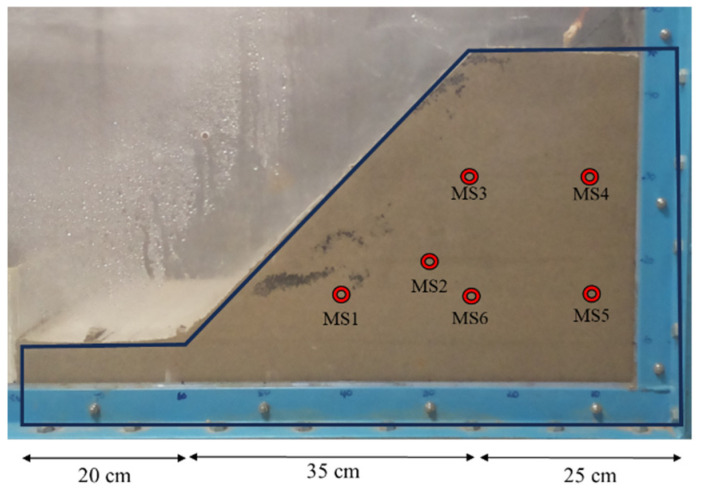
Overview of slope model and moisture sensor layout.

Slope model experiment was designed to test the soil moisture sensors in a controlled environment under rainfall intensities of 100, 70, and 45 mm/h. The dimensions of the slope model and the positions of the sensors for the experiments are illustrated in Figure 5. The slope model measured 80 cm in length, 40 cm in width, and 45 cm in height. The slope angle was set to 45°. This slope angle was chosen as it represents a practical compromise between stability and the risk of failure, allowing for effective modeling of real-world conditions. Six moisture sensors (MS) were embedded within the slope to measure the soil moisture contents in different areas to assess moisture distribution during rainfall events. The sensors (MS1 to MS6) were positioned to capture variations in soil moisture at various depths and horizontal locations across the slope. The slope model was constructed by compacting the soil every 5 cm, while the base layer was compacted at 10 cm of height. There were a total of eight soil layers with a soil dry density of 1.40 g/cm^3^ and an initial water content of 10%. A rainfall simulator was designed to simulate uniform and continuous rainfall with different rainfall intensities. The rainfall was generated by nozzles attached to the rainfall simulator frame installed above the flume tank.

After each experiment, soil samples were collected from the near respective moisture sensors. The purpose is to measure the actual moisture contents ($w_{act}$) of the soil and verify the result with the moisture contents monitored by the sensors. Moisture content from the collected soil samples were measured by oven drying them. It is expressed by the weight ratio difference between wet and dry samples. The water mass ratio is established by drying the soil samples in an oven at a temperature of 100–110 °C until a constant mass is reached, followed by measuring the weight of the soil samples before and after the drying process. The calculation is the following formula:

(1) $$w_{act}=\frac{m_{a}-m_{b}}{m_{b}-m_{c}}\times 100$$

where $m_{a}$ is mass of the sample and container, $m_{b}$ is mass of the oven-dried sample and container, and $m_{c}$ is mass of container in grams.

### 2.4. Statistical Evaluation

Three statistical indicators were used to quantify the accuracy of the calibration equations: coefficient of determination (*R*^2^), root mean square error (RMSE), and mean biased error (MBE) [24,36]. *R*^2^ describes the correlation between the calibrated and actual values (Equation (2)), RMSE was used to describe the accuracy between calibrated and actual values (Equation (3)), and mean biased error (MBE) was used to measure the deviation of overall calibration results (Equation (4)).

(2) $$\;R^{2}=\frac{{\left[\sum_{i=1}^{n}\left(w_{s}-\bar{w_{s}}\right)\left(w_{act}-\bar{w_{act}}\right)\right]}^{2}}{\sum_{i=1}^{n}{\left(w_{s}-\bar{w_{s}}\right)}^{2}\times \sum_{i=1}^{n}{\left(w_{act}-\bar{w_{act}}\right)}^{2}}$$

(3) $$RMSE=\sqrt{\frac{1}{n}\overset{n}{\underset{i=1}{\sum }}{\left(w_{s}-w_{act}\right)}^{2}}$$

(4) $$MBE=\frac{1}{n}\overset{n}{\underset{i=1}{\sum }}\left(w_{s}-w_{act}\right)$$

where n is the sample size, $w_{s}$ is the sensor measured value, $\bar{w_{s}}$ is the mean sensor measured value, $w_{act}$ is the actual value, and $\bar{w_{act}}$ is the mean actual value.

## 3. Results

### 3.1. Moisture Sensor Calibration

The SKU:SEN0193 is one of the moisture sensors designed for integration into the Internet of Things (IoT), allowing for operation at low power ranging from 3.3 to 5 V. Verifying the operating voltage of 3.3 to 5 V in the moisture sensor, including checking the timer chips and voltage regulator [37,38] was the initial step before utilizing it. In this study, the authors were using SKU:SEN0193 v1.0. Both the timer chip and voltage regulator for all moisture sensors were examined and found to operate within the 3.3 to 5 V range.

**Figure 6:**
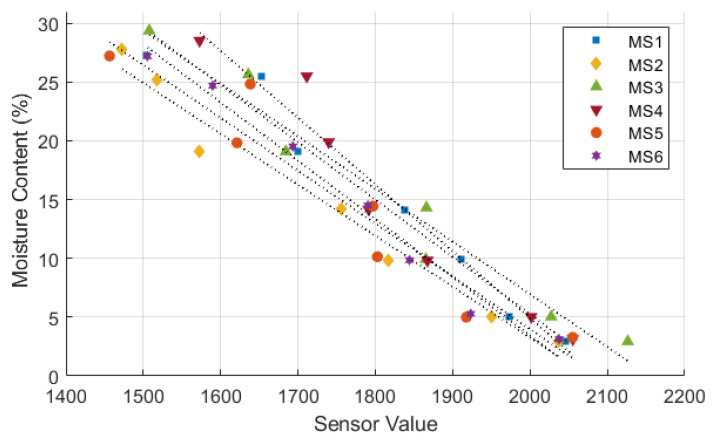
Calibration of moisture sensor using a linear equation.

**Figure 7:**
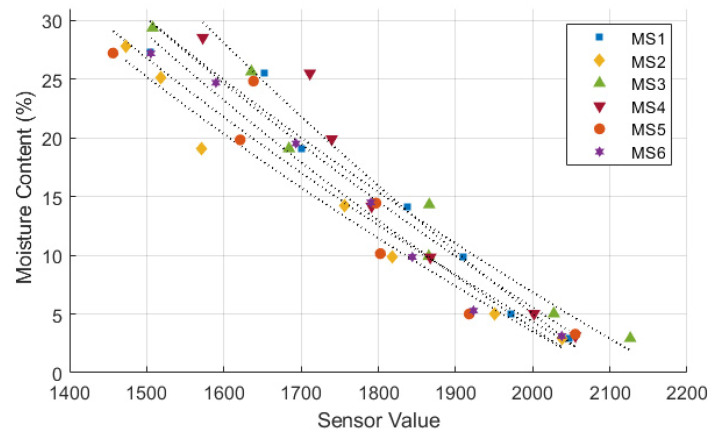
Calibration of moisture sensor using logarithmic equation.

**Figure 8:**
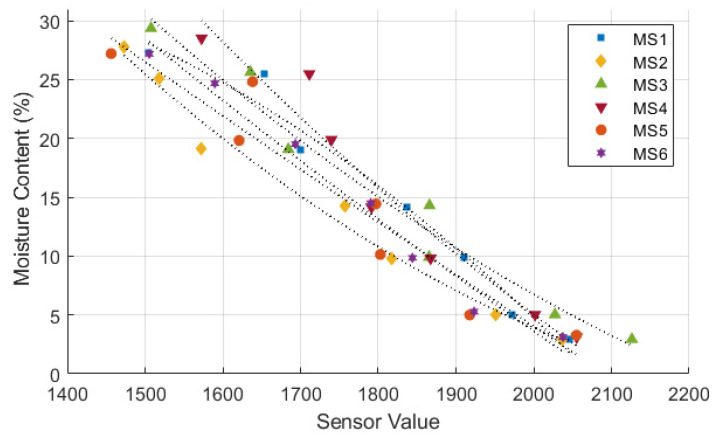
Calibration of moisture sensor using polynomial equation.

Figure 6, Figure 7 and Figure 8 illustrate the sensor values obtained from all tested moisture sensors plotted against true soil moisture contents, and the fitting curves of three different types of calibration equations are shown as dotted lines. True soil moisture content refers to the actual moisture content measured from oven-dried samples. The calibration lines closely align for all sensors, indicating consistent performance across different sensor units in measuring soil moisture content. Each sensor demonstrates a similar trend but with variations in sensor value readings, suggesting minor differences in calibration or sensitivity among the sensors. The calibration equation results and statistical evaluation of moisture sensors are presented in Table 3. Statistical analysis reveals that a polynomial equation yields the best calibration results due to its high *R*^2^ and low RMSE compared to other type of equations. Furthermore, data from the calibration process follows a polynomial trend, which can be attributed to a nonlinear behavior of the sensor’s response to changes in soil moisture [21,22,39,40].

**Figure 9:**
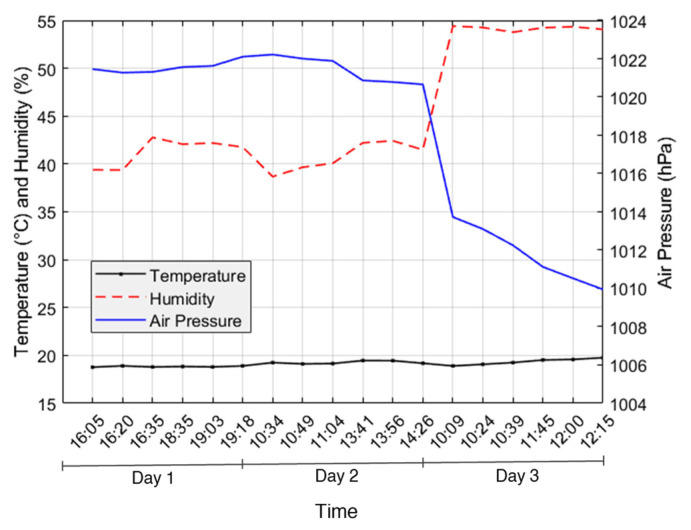
Room temperature, humidity and air pressure during the calibration process.

Room temperature, relative humidity and air pressure during the calibration process were shown in Figure 9 and presented as text axis, which displays label as categories along the x-axis. Calibration process took up three days due to technical issues in the preparation. The room temperature is relatively constant during the calibration process, ranging from 18.7 to 19.7 °C. Relative humidity shows an increase, while air pressure decreases over time with a significant drop around the point where humidity sharply rises. This suggests that humidity and air pressure have an inverse relationship; humidity tends to rise as air pressure declines, likely because lower pressure permits the air to contain more moisture. Temperature appears to be less directly affected by these changes, maintaining a consistent trend despite the variations in humidity and air pressure.

**Figure 10:**
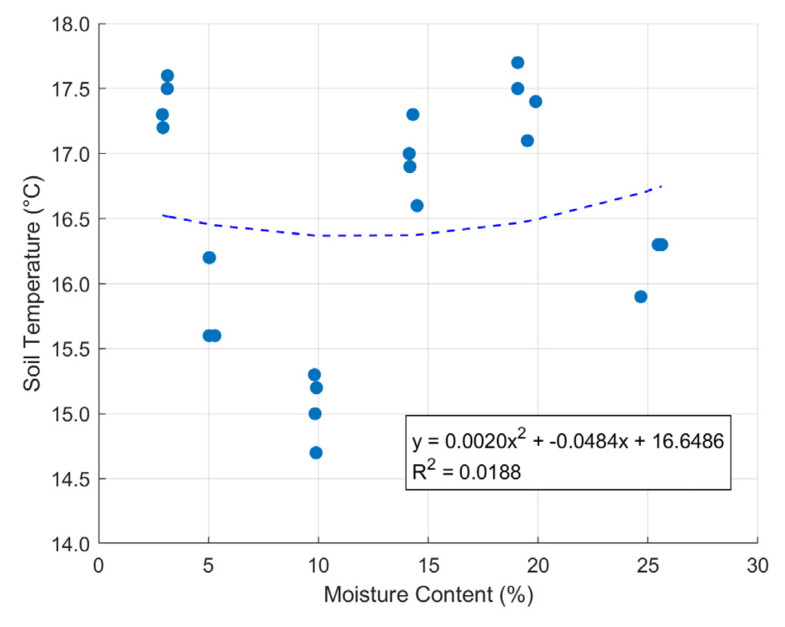
Moisture content under soil temperature variation.

Figure 10 illustrates the relationship between soil moisture content and soil temperature, with a trendline fitted using a quadratic regression model. Soil temperatures were measured near moisture sensors 1 3, 4, and 6. The data points are scattered, suggesting a lack a weak correlation between these variables. The fitted quadratic equation has an *R*^2^ value of 0.0188, indicating that only a small portion of the variance in moisture content is explained by changes in soil temperature. The trendline shows a slight upward curvature, which implies a minimal increase in temperature as moisture content increases, but the low *R*^2^ value suggests this relationship is weak and may not be statistically significant.

### 3.2. Moisture Content in Slope Models

In slope model experiments, it is essential to assess the fundamental aspects of slope behavior and evaluate the roles played by factors such as geometry, initial and boundary conditions, and soil properties. However, since this study aims to validate the results of moisture sensor calibration equations, the aforementioned aspects are not the focus of this study.

**Figure 11:**
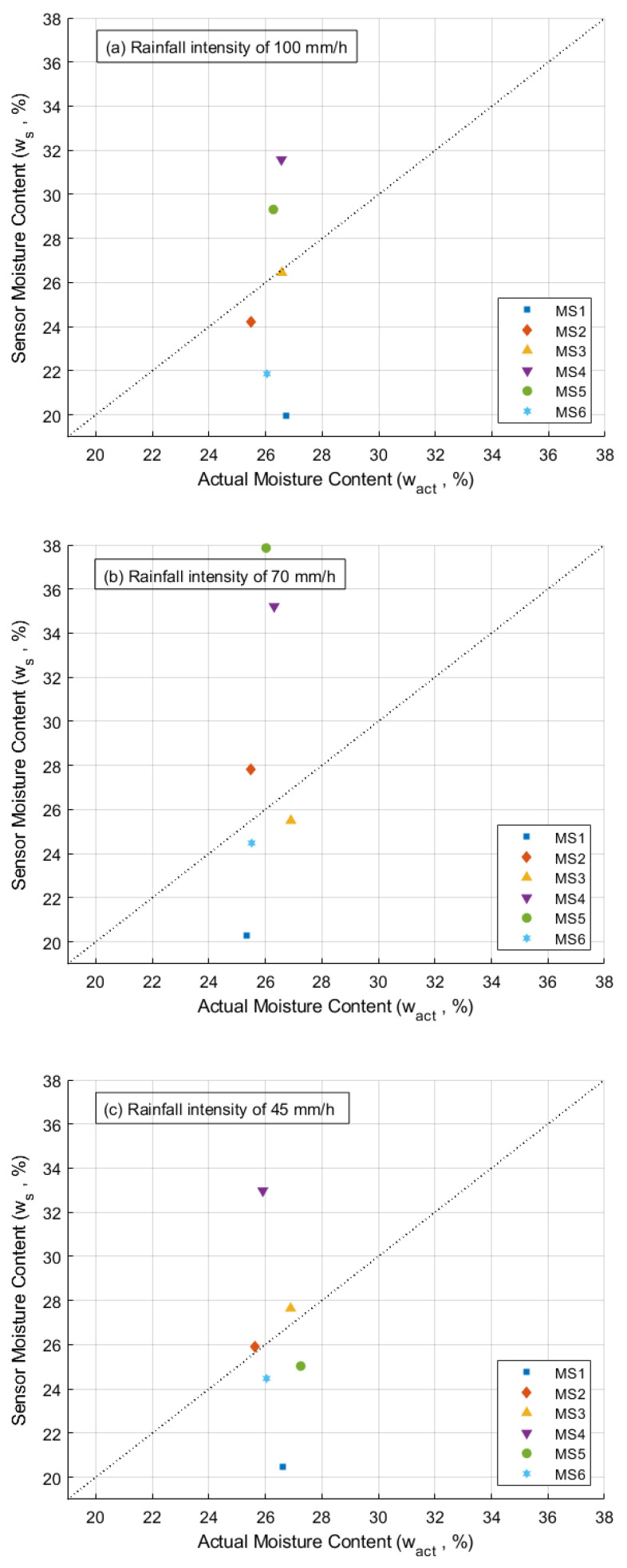
Comparison of sensor moisture content with actual moisture content on different rainfall intensities.

In this section, moisture sensors were tested in slope model experiments, and the soil moisture contents were observed under different artificial rainfall conditions. After the slope model experiment concluded, soil samples were collected to measure the actual soil moisture content ($w_{act}$), which was then compared to the moisture content readings obtained by the sensors $w_{s}$. Figure 11 presents the comparison of sensor moisture content with actual moisture content on different rainfall intensities. The data points show deviations from the 1:1 dotted line, highlighting variations in sensor readings compared to actual moisture content. As an example, MS4 in the end of slope model experiment with rainfall intensity of 100 mm/h recorded moisture content of 31.6%, while the actual moisture content was 26.6%. This indicates that MS4 overestimating the moisture content within the soil. Similarly, MS1 recorded value of 20.0%, while the actual moisture content was 26.7%, showing an underestimation of actual moisture content. The differences in moisture contents (Δw) between the sensor and actual are presented in Table 4. Positive values indicate an overestimation of moisture content, while negative values indicate an underestimation of moisture content by the sensors. The mean bias error of the sensors in the slope model experiment under rainfall intensities of 100, 70, and 45 mm/h are −0.72, 2.61, and −0.30, respectively.

**Figure 12:**
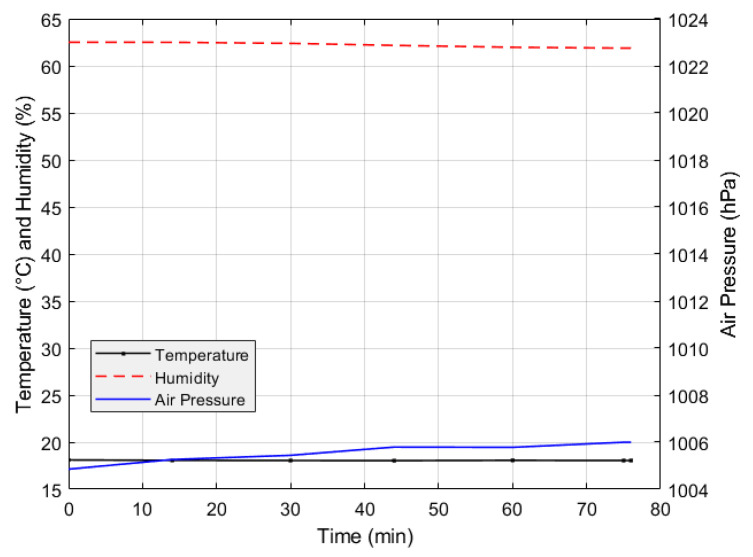
Temperature, humidity and air pressure during slope model experiment (100 mm/h).

During the slope model experiment, temperature, humidity and air pressure has minor changes, as presented in Figure 12. The temperature remains within a narrow range, with a difference of only 0.06 °C, indicating a stable trend. Humidity levels are also stable, consistently around 62%, indicating no significant changes in moisture levels. Air pressure shows slight increase but remains relatively steady, above 1004 hPa. This stability shows that the environmental conditions during the slope model experiment remained consistent.

## 4. Discussion

### 4.1. Calibration Evaluation

**Figure 13:**
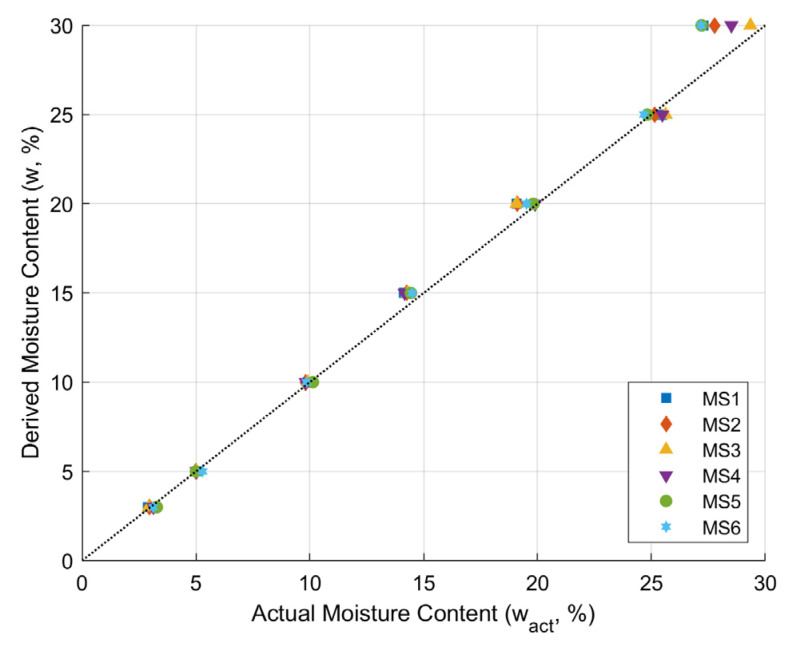
Comparison of derived moisture content with actual moisture content during the calibration process.

Performance evaluation of soil moisture sensors was conducted in laboratory settings. Soil temperature during sensor calibration was constant at 16.0 ± 1.4 °C. The comparison of moisture content with actual moisture content is presented in Figure 13. The derived moisture contents (w) were obtained from the known quantities of water added to the oven-dried soil before the calibration process, while the actual moisture contents ($w_{act}$) were determined by the oven-drying soil samples after the measurement in the calibration process. It is shown that the derived moisture and the actual moisture content are almost identical. However, the values changed when the moisture content exceeded 25%, suggesting that the recorded moisture content deviates when the soil reaches critical condition.

Statistical results reveal that individual sensor calibration using a polynomial equation yielded the lowest *R*^2^ of 0.918 and the highest RMSE of 2.488, while the universal or generalized calibration from all sensors produces values of *R*^2^ and RMSE of 0.901 and 2.554, respectively. The universal calibration equation was derived by incorporating data from all individual moisture sensor calibrations, plotting them on a graph and estimating its *R*^2^ and RMSE. Despite the slightly lower value of *R*^2^, the universal calibration exhibited higher RMSE values. A high RMSE value indicates a significant deviation between the sensor-measured moisture contents and the actual moisture contents, indicating a lack of precision in the model. Therefore, although individual sensor calibrations demonstrated slightly higher *R*^2^ values compared to universal calibrations, the higher RMSE values in the universal calibration highlight greater imprecision and systematic overestimation errors. It is important to note that converting the raw sensor values using universal calibration can lead to inaccuracies when measuring the initial moisture content, despite the consistent trend of increasing moisture content. These inaccuracies are likely due to the inherent variability of individual sensors. Each sensor may have unique characteristics and responses due to manufacturing differences, environmental conditions, and wear and tear. Universal calibration applies a generalized correction that cannot accommodate these individual sensor variances, resulting in systematic error and inaccuracies.

This finding is specific to the tested soil sample and may have different results with different types of soil and conditions [41,42]. Previous studies [39,43,44] have consistently emphasized the importance of utilizing a local or individual calibration curve for a specific type of soil rather than a universal calibration approach. The polynomial function of the individual sensor calibration curve demonstrates satisfactory consistency between soil moisture content and sensor value with *R*^2^ greater than 0.90, similar to the correlation found in other studies [22,27,43,45].

### 4.2. Sensor Performance in Slope Model

**Figure 14:**
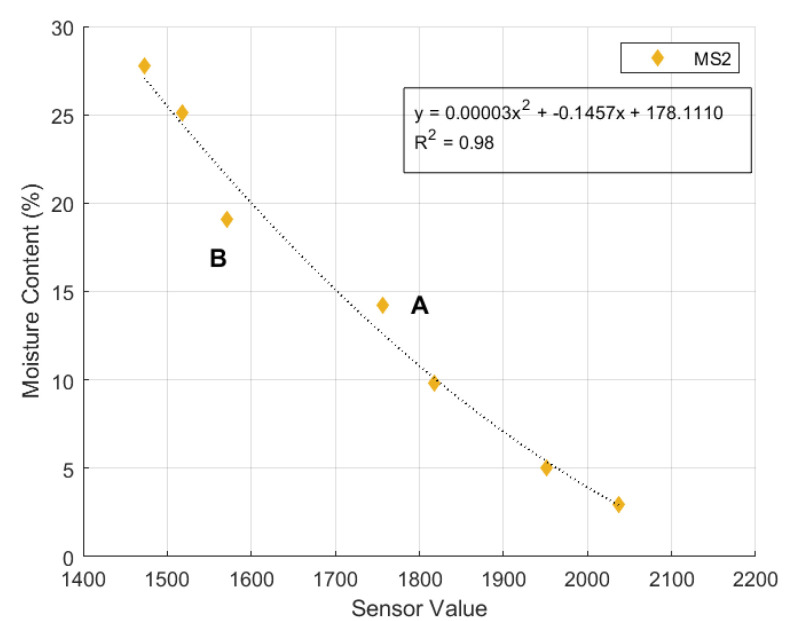
Calibration equation of Moisture Sensor number 2.

In the end of slope model experiments, soil samples near moisture sensors were collected to measure the actual soil moisture content. Moisture content from the sensors are varies compared to actual moisture content, as presented by the data points diverging from the 1:1 reference line in Figure 11. The disparity between the actual and the sensor moisture content (Δw) could be caused by the calibration process and its estimations. For instance, the calibration data of moisture sensor number 2 (MS2) is illustrated in Figure 14. At point A, the polynomial equation underestimates the mois-ture content. During calibration, a sensor value of 1757 corresponded to 14.2% of the soil moisture content. However, when using the individual calibration equation for MS2, the same sensor value is equal to 12.5% of moisture content.

On the contrary, the calibration equation overestimates the soil moisture content value at point B. During the calibration process, a sensor value of 1572 corresponded to a soil moisture content of 19.1%, while the calculated moisture content using the poly-nomial calibration equation was 21.5%. The deviation in sensor readings could reduce the accuracy of the monitoring system. However, in the case of an example presented above, the moisture content difference or error of ±12 % is acceptable given that previous research has already mentioned that this sensor has medium accuracy [43]. It is important to note that both underestimation and overestimation occur in all moisture sensors, as presented in Figure 8.

The calibration process and the slope model experiment were carried out around the end of winter, which explains the relatively low temperatures observed. During the calibration process, variations in humidity and air pressure were observed. However, these variables slightly changes during the slope model experiment. This difference can be attributed to the timing of the two experiments: sensor calibration was performed at different times over three days, when air pressure and relative humidity naturally fluctuate, while the slope model experiment was conducted in the middle of the night, when the environmental conditions were more stable [46,47]. Following the noted differences in environmental conditions during the calibration process, soil temperature shows a weak correlation in its response to varying moisture contents, suggesting that it may not serve as a reliable predictor of moisture content in this setup. Moreover, the relatively neutral pH of the K7 sand used in the study was considered stable for calibration purposes, and given the narrow temperature range in our controlled environment, any influence from temperature was deemed minimal for this study. Overall, these findings emphasize the importance of timing in experimental design, as it significantly impacts the environmental conditions affecting sensor calibration and performance.

The drawbacks of the experiment included, but were not limited to, experimental and human errors, as well as the use of soil with uniform particle size (Figure 4). Uniform soil, such as coarse sand, has a lower sensor–surface contact area, while fine sand has a higher sensor–surface contact area, both of which affect sensor readings. Additionally, soil temperature, salinity, and bulk density also influence sensor values [48,49]. Another aspect that was not tested in this research is the durability of the sensor under laboratory conditions. To improve the accuracy of sensor readings and mitigate these issues, several methods can be implemented: (1) Conducting calibration process with different soil particle size, salinity and density; (2) Periodic calibration is crucial, as sensor age can affect reliability [50]. The calibration of sensor systems, like the interdigital conductance sensor for measuring liquid film thickness, highlights the importance of accurate measurements for system reliability [51]; (3) Conducting calibration and slope model experiments in similar environmental settings to improve precision, and; (4) Applying filters such as the Kalman filter helps reduce noise and correct systematic errors in sensor data [52]. Furthermore, utilizing machine learning algorithms enables analysis of sensor drift, noise and biases over time [53,54].

In this initial study, the calibration was designed to capture soil-specific characteristics. However, this may not fully represent all variables affecting sensor accuracy, particularly under varied slope conditions. Further study is necessary, including data collection for calibration under various types of soil, temperature, salinity, bulk density, pH, and other environmental parameters relevant to slope stability scenarios. Additionally, expanding the study to validate the calibration and accuracy of soil moisture sensors by deploying the system in the field to monitor an actual slope will enhance understanding of sensor behavior under complex conditions. This validation will provide insights into data stability, sensor durability, lifespan, and other factors that may contribute to data faults [50]. For instance, moisture sensors deployed in the field may experience higher noise compared to those in a laboratory study [55]. Therefore, denoising the transmitted data received by the server is important to avoid false alarms in the development of the landslide early warning system. Finally, the authors would suggest maintaining the total construction of the monitoring system as minimal as possible since the system has the potential to be adapted as a landslide early warning system installed across the globe and reduce the impact of disasters caused by landslides.

## 5. Conclusions

To ensure the accuracy and reliability of low-cost moisture sensors, calibration for specific soil types and properties is important, as calibration results significantly influence sensor readings. This study evaluated the performance of six low-cost soil moisture sensor (SKU: SEN1093) through laboratory experiments. The study began with the calibration of moisture sensors to develop calibration equations and identify the equation that yields the most accurate results. The calibration results were then verified through three slope model experiments using silica sand under varying rainfall intensities. 

The polynomial calibration equation demonstrated the highest accuracy, with the lowest *R*^2^ of 0.918, the highest RMSE of 2.488, compared to the linear and logarithmic equations. This makes the polynomial calibration equation the most reliable model for predicting soil moisture content among the tested equations. In addition, the study found that individual sensor calibration achieved more consistent accuracy than universal calibration, with individual sensors demonstrating prediction errors within an acceptable range (±12%) for practical applications. Furthermore, calibration stability was affected by environmental conditions, even within the relatively stable, controlled setting of this study. Conducting sensor calibration in controlled environments with stable factors, such as room temperature, humidity, air pressure, and soil temperature, is essential to establish a baseline of reliability. Although real-world conditions vary, such calibration provides a foundation to account for environmental fluctuations in landslide early warning systems, where consistent sensor performance is crucial.

The integration of low-cost moisture sensors with the Internet of Things (IoT) demonstrates significant potential for developing landslide early warning systems. In-depth slope model research that incorporates all parameters affecting sensor behavior under various conditions, such as soil temperature, salinity, particle size, and bulk density, is necessary to optimize system accuracy. Additionally, further studies are needed to evaluate sensor performance in real-world field conditions, ensuring long-term reliability of the system and effectiveness for landslide mitigation applications.

## Figures and Tables

**Table 1:** The overall expenditure associated with the implementation of the soil moisture monitoring system (US$ in May 2024).

Item	Quantity	Unit Cost ($)	Subtotal ($)	Total ($)
M5Stack Core2	3	46.90	140.7	
SKU:SEN0193	6	5.90	35.4	
Slyfox	2	39.15	78.3	
ENV III sensor	1	5.95	5.95	
				260.35

**Table 2:** Soil properties of K7 sand.

Description	Value
Specific Gravity, *Gs*	2.62
Density of soil, *ρ*_t_ (g/cm^3^)	1.51
Dry density of soil, *ρ*_d_ (g/cm^3^)	1.40
Maximum dry density of soil, *ρ*_d max_ (g/cm^3^)	1.60
Minimum dry density of soil, *ρ*_d min_ (g/cm^3^)	1.19
Void ratio, *e*	0.87

**Table 3:** Calibration equation results and statistics evaluation of moisture sensors.

Sensor	Equation Type	Calibration Equation	*R* ^2^	RMSE
MS1	Linear	y = −0.0487x + 102.6980	0.964	1.672
	Logarithmic	y = −85.5833 log(x) + 656.0471	0.954	1.911
	Polynomial	y = −3 × 10^−5^x^2^ + 0.0621x + 5.1443	0.974	1.429
MS2	Linear	y = −0.0433x + 89.9223	0.973	1.463
	Logarithmic	y = −75.3425 log(x) + 576.1804	0.980	1.270
	Polynomial	y = 3 × 10^−5^x^2^ + −0.1457x + 178.1110	0.983	1.171
MS3	Linear	y = −0.0448x + 96.4779	0.960	1.857
	Logarithmic	y = −80.9699 log(x) + 622.2928	0.966	1.725
	Polynomial	y = 2 × 10^−5^x^2^ + −0.1211x + 165.0332	0.967	1.687
MS4	Linear	y = −0.0568x + 118.4671	0.943	2.186
	Logarithmic	y = −103.2243 log(x) + 789.6224	0.945	2.143
	Polynomial	y = 2 × 10^−5^x^2^ + −0.1346x + 188.9894	0.946	2.128
MS5	Linear	y = −0.0449x + 93.7915	0.918	2.489
	Logarithmic	y = −78.0259 log(x) + 597.4044	0.915	2.534
	Polynomial	y = 2 × 10^−5^x^2^ + −0.0534x + 101.1744	0.918	2.488
MS6	Linear	y = −0.0495x + 102.5089	0.980	1.231
	Logarithmic	y = −86.8725 log(x) + 664.1057	0.979	1.245
	Polynomial	y = 1 × 10^−5^x^2^ + −0.0756x + 125.3305	0.980	1.213

**Table 4:** The difference between sensor and actual moisture content on different rainfall intensities.

Sensor	Rainfall Intensity of 100 mm/h			Rainfall Intensity of 70 mm/h			Rainfall Intensity of 45 mm/h		
	$w_{s}\;$ (%)	$w_{act}\;$ (%)	Δw (%)	$w_{s}\;$ (%)	$w_{act}\;$ (%)	Δw (%)	$w_{s}\;$ (%)	$w_{act}\;$ (%)	Δw (%)
MS1	20.0	26.7	−6.7	20.3	25.3	−5.0	20.5	26.6	−6.1
MS2	24.2	25.5	−1.3	27.8	25.5	2.3	25.9	25.6	0.3
MS3	26.5	26.6	−0.1	25.5	26.9	−1.4	27.6	26.9	0.7
MS4	31.6	26.6	5.0	35.2	26.3	8.9	33.0	25.9	7.1
MS5	29.3	26.3	3.0	37.9	26.0	11.9	25.0	27.3	−2.3
MS6	21.9	26.0	−4.1	24.5	25.5	−1.0	24.5	26.0	−1.5

## Data Availability

Data available on request.

## References

[1] Froude M.J., Petley D.N. (2018). Global Fatal Landslide Occurrence from 2004 to 2016. Nat. Hazards Earth Syst. Sci..

[2] Saito H., Korup O., Uchida T., Hayashi S., Oguchi T. (2014). Rainfall Conditions, Typhoon Frequency, and Contemporary Landslide Erosion in Japan. Geology.

[3] Schuster R.L., Fleming R.W. (1986). Economic Losses and Fatalities Due to Landslides. Environ. Eng. Geosci..

[4] Guzzetti F., Gariano S.L., Peruccacci S., Brunetti M.T., Marchesini I., Rossi M., Melillo M. (2020). Geographical Landslide Early Warning Systems. Earth Sci. Rev..

[5] Sorensen J.H. (2000). Hazard Warning Systems: Review of 20 Years of Progress. Nat. Hazards Rev..

[6] Ahmadi-adli M., Huvaj N., Toker N.K. (2017). Rainfall-Triggered Landslides in an Unsaturated Soil: A Laboratory Flume Study. Environ. Earth Sci..

[7] Xu J., Ueda K., Uzuoka R. (2022). Evaluation of Failure of Slopes with Shaking-Induced Cracks in Response to Rainfall. Landslides.

[8] Chueasamat A., Hori T., Saito H., Sato T., Kohgo Y. (2018). Experimental Tests of Slope Failure Due to Rainfalls Using 1g Physical Slope Models. Soils Found..

[9] Liu Y., Hazarika H., Kanaya H., Takiguchi O., Rohit D. (2023). Landslide Prediction Based on Low-Cost and Sustainable Early Warning Systems with IoT. Bull. Eng. Geol. Environ..

[10] Hidayat M.N., Hazarika H., Fujishiro T., Murai M., Manandhar S., Liu Y., Fukumoto Y., Kanaya H., Takiguchi O., Hazarika H., Haigh S.K., Chaudhary B., Murai M., Manandhar S. (2024). Evaluation of Landslide Triggering Mechanism During Rainfall in Slopes Containing Vertical Cracks. Geo-Sustainnovation for Resilient Society.

[11] Cogan J., Gratchev I. (2019). A Study on the Effect of Rainfall and Slope Characteristics on Landslide Initiation by Means of Flume Tests. Landslides.

[12] Huang C.-C., Yuin S.-C. (2010). Experimental Investigation of Rainfall Criteria for Shallow Slope Failures. Geomorphology.

[13] Pajalić S., Peranić J., Maksimović S., Čeh N., Jagodnik V., Arbanas Ž. (2021). Monitoring and Data Analysis in Small-Scale Landslide Physical Model. Appl. Sci..

[14] Peranić J., Čeh N., Arbanas Ž. (2022). The Use of Soil Moisture and Pore-Water Pressure Sensors for the Interpretation of Landslide Behavior in Small-Scale Physical Models. Sensors.

[15] Zhao B., Dai Q., Zhuo L., Zhu S., Shen Q., Han D. (2021). Assessing the Potential of Different Satellite Soil Moisture Products in Landslide Hazard Assessment. Remote Sens. Environ..

[16] Segoni S., Rosi A., Lagomarsino D., Fanti R., Casagli N. (2018). Brief Communication: Using Averaged Soil Moisture Estimates to Improve the Performances of a Regional-Scale Landslide Early Warning System. Nat. Hazards Earth Syst. Sci..

[17] Ray R.L., Jacobs J.M. (2007). Relationships among Remotely Sensed Soil Moisture, Precipitation and Landslide Events. Nat. Hazards.

[18] Rawat P.S., Barthwal A. (2024). Landslide Monitor: A Real-Time Landslide Monitoring System. Environ. Earth Sci..

[19] Oguz E.A., Depina I., Myhre B., Devoli G., Rustad H., Thakur V. (2022). IoT-Based Hydrological Monitoring of Water-Induced Landslides: A Case Study in Central Norway. Bull. Eng. Geol. Environ..

[20] Placidi P., Vergini C.V.D., Papini N., Cecconi M., Mezzanotte P., Scorzoni A. (2023). Low-Cost and Low-Frequency Impedance Meter for Soil Water Content Measurement in the Precision Agriculture Scenario. IEEE Trans. Instrum. Meas..

[21] López E., Vionnet C., Ferrer-Cid P., Barcelo-Ordinas J.M., Garcia-Vidal J., Contini G., Prodolliet J., Maiztegui J. (2022). A Low-Power IoT Device for Measuring Water Table Levels and Soil Moisture to Ease Increased Crop Yields. Sensors.

[22] Kulmány I.M., Bede-Fazekas Á., Beslin A., Giczi Z., Milics G., Kovács B., Kovács M., Ambrus B., Bede L., Vona V. (2022). Calibration of an Arduino-Based Low-Cost Capacitive Soil Moisture Sensor for Smart Agriculture. J. Hydrol. Hydromech..

[23] Borah S., Kumar R., Mukherjee S. (2020). Low-Cost IoT Framework for Irrigation Monitoring and Control. Int. J. Intell. Unmanned Syst..

[24] Nagahage E.A.A.D., Nagahage I.S.P., Fujino T. (2019). Calibration and Validation of a Low-Cost Capacitive Moisture Sensor to Integrate the Automated Soil Moisture Monitoring System. Agriculture.

[25] Faia P.M., Libardi J., Barbosa I., Araújo E.S., de Oliveira H.P. (2017). Preparation, Characterization, and Evaluation of Humidity-Dependent Electrical Properties of Undoped and Niobium Oxide-Doped TiO_2_: WO_3_ Mixed Powders. Adv. Mater. Sci. Eng..

[26] Mendes S., Kurapova O., Faia P., Pazheltsev V., Zaripov A., Konakov V. (2023). Polyantimonic Acid-Based Materials Evaluated as Moisture Sensors at Ambient Temperature. J. Solid. State Electrochem..

[27] Pereira R.M., Sandri D., Silva Júnior J.J. (2022). da Evaluation of Low-Cost Capacitive Moisture Sensors in Three Types of Soils in the Cerrado, Brazil. Rev. Eng. Na Agric.-REVENG.

[28] DFRobot Capacitive Soil Moisture Sensor SKU:SEN0193. https://wiki.dfrobot.com/Capacitive_Soil_Moisture_Sensor_SKU_SEN0193.

[29] Slyfox 3 in 1 Soil Moisture Meter for Agriculture. https://www.amazon.co.jp/-/en/dp/B0C5SWTL78/?coliid=IMXG1A10B6K1R&colid=G4CJWLMA6XDT&psc=1&ref_=list_c_wl_lv_ov_lig_dp_it.

[30] Tang J., Taro U., Huang D., Xie J., Tao S. (2020). Physical Model Experiments on Water Infiltration and Failure Modes in Multi-Layered Slopes under Heavy Rainfall. Appl. Sci..

[31] Lourenço S.D.N., Sassa K., Fukuoka H. (2006). Failure Process and Hydrologic Response of a Two Layer Physical Model: Implications for Rainfall-Induced Landslides. Geomorphology.

[32] Wang G., Sassa K. (2001). Factors Affecting Rainfall-Induced Flowslides in Laboratory Flume Tests. Géotechnique.

[33] Wang F., Dai Z., Takahashi I., Tanida Y. (2020). Soil Moisture Response to Water Infiltration in a 1-D Slope Soil Column Model. Eng. Geol..

[34] Placidi P., Gasperini L., Grassi A., Cecconi M., Scorzoni A. (2020). Characterization of Low-Cost Capacitive Soil Moisture Sensors for IoT Networks. Sensors.

[35] Domínguez-Niño J.M., Bogena H.R., Huisman J.A., Schilling B., Casadesús J. (2019). On the Accuracy of Factory-Calibrated Low-Cost Soil Water Content Sensors. Sensors.

[36] Datta S., Taghvaeian S., Ochsner T., Moriasi D., Gowda P., Steiner J. (2018). Performance Assessment of Five Different Soil Moisture Sensors under Irrigated Field Conditions in Oklahoma. Sensors.

[37] Voltage Regulator—XC6206P302MR (65Z5). https://www.sunrom.com/p/xc6206p302mr-65z5.

[38] Texas Instruments (2019). TLC555 LinCMOSTM Timer Chip.

[39] Bircher S., Andreasen M., Vuollet J., Vehviläinen J., Rautiainen K., Jonard F., Weihermüller L., Zakharova E., Wigneron J.-P., Kerr Y.H. (2016). Soil Moisture Sensor Calibration for Organic Soil Surface Layers. Geosci. Instrum. Methods Data Syst..

[40] Radi, Murtiningrum, Ngadisih, Muzdrikah F.S., Nuha M.S., Rizqi F.A. (2018). Calibration of Capacitive Soil Moisture Sensor (SKU:SEN0193). Proceedings of the 2018 4th International Conference on Science and Technology (ICST).

[41] Dong Y., Miller S., Kelley L. (2020). Performance Evaluation of Soil Moisture Sensors in Coarse- and Fine-Textured Michigan Agricultural Soils. Agriculture.

[42] Li B., Wang C., Gu X., Zhou X., Ma M., Li L., Feng Z., Ding T., Li X., Jiang T. (2022). Accuracy Calibration and Evaluation of Capacitance-Based Soil Moisture Sensors for a Variety of Soil Properties. Agric. Water Manag..

[43] Schwamback D., Persson M., Berndtsson R., Bertotto L.E., Kobayashi A.N.A., Wendland E.C. (2023). Automated Low-Cost Soil Moisture Sensors: Trade-Off between Cost and Accuracy. Sensors.

[44] Campora M., Palla A., Gnecco I., Bovolenta R., Passalacqua R. (2020). The Laboratory Calibration of a Soil Moisture Capacitance Probe in Sandy Soils. Soil. Water Res..

[45] Adla S., Rai N.K., Karumanchi S.H., Tripathi S., Disse M., Pande S. (2020). Laboratory Calibration and Performance Evaluation of Low-Cost Capacitive and Very Low-Cost Resistive Soil Moisture Sensors. Sensors.

[46] Howell J.F., Sun J. (1999). Surface-Layer Fluxes in Stable Conditions. Bound. Layer. Meteorol..

[47] Hughes M., Hall A., Fovell R.G. (2007). Dynamical Controls on the Diurnal Cycle of Temperature in Complex Topography. Clim. Dyn..

[48] Chandel A., Swami D., Joshi N. (2024). Calibration Complexities: Full-Scale Error Impact and Simultaneous Variation of Salinity, Temperature, and Moisture Content on Sensor Performance in Soil. Env. Dev. Sustain..

[49] Seyfried M.S., Murdock M.D. (2001). Response of a New Soil Water Sensor to Variable Soil, Water Content, and Temperature. Soil Sci. Soc. Am. J..

[50] Ni K., Ramanathan N., Chehade M.N.H., Balzano L., Nair S., Zahedi S., Kohler E., Pottie G., Hansen M., Srivastava M. (2009). Sensor Network Data Fault Types. ACM Trans. Sens. Netw..

[51] Ren W., Jin N., Wang T. (2024). An Interdigital Conductance Sensor for Measuring Liquid Film Thickness in Inclined Gas–Liquid Two-Phase Flow. IEEE Trans. Instrum. Meas..

[52] Park S., Gil M.-S., Im H., Moon Y.-S. (2019). Measurement Noise Recommendation for Efficient Kalman Filtering over a Large Amount of Sensor Data. Sensors.

[53] Schmidt J.Q., Kerkez B. (2023). Machine Learning-Assisted, Process-Based Quality Control for Detecting Compromised Environmental Sensors. Environ. Sci. Technol..

[54] Pereira M., Glisic B. (2023). Detection and Quantification of Temperature Sensor Drift Using Probabilistic Neural Networks. Expert. Syst. Appl..

[55] Hidayat M.N., Hazarika H., Murai M., Kanaya H. Application of Early Warning System for Monitoring Landslide Vulnerability of Slope. Proceedings of the 7th International Conference on Geotechnics, Civil Engineering and Structures, CIGOS.

